# Nine patients with *KCNQ2*-related neonatal seizures and functional studies of two missense variants

**DOI:** 10.1038/s41598-023-29924-y

**Published:** 2023-02-27

**Authors:** Suphalak Chokvithaya, Natarin Caengprasath, Aayalida Buasong, Supavadee Jantasuwan, Kanokwan Santawong, Netchanok Leela-adisorn, Siraprapa Tongkobpetch, Chupong Ittiwut, Vitchayaporn Emarach Saengow, Wuttichart Kamolvisit, Ponghatai Boonsimma, Saknan Bongsebandhu-phubhakdi, Vorasuk Shotelersuk

**Affiliations:** 1 Center of Excellence for Medical Genomics, Medical Genomics Cluster, Department of Pediatrics, Faculty of Medicine, Chulalongkorn University, Bangkok, 10330 Thailand; 2 Excellence Center for Genomics and Precision Medicine, King Chulalongkorn Memorial Hospital, The Thai Red Cross Society, Bangkok, 10330 Thailand; 3 Department of Clinical Pathology and Medical Technology Laboratory, Queen Sirikit National Institute of Child Health, Ministry of Public Health, Bangkok, Thailand; 4 Department of Physiology, Faculty of Medicine, Chulalongkorn University, Bangkok, 10330 Thailand; 5 Chula Neuroscience Center, King Chulalongkorn Memorial Hospital, The Thai Red Cross Society, Bangkok, Thailand; 6 Department of Pediatrics, Maharat Nakhon Ratchasima Hospital, Nakhon Ratchasima, Thailand; 7 Department of Stem Cell and Cell, Therapy Research Unit, Faculty of Medicine, Chulalongkorn University, Bangkok, Thailand

**Keywords:** Genetics, Functional genomics, Medical genetics, Neurodevelopmental disorders

## Abstract

Mutations in *KCNQ2* encoding for voltage-gated K channel subunits underlying the neuronal M-current have been associated with infantile-onset epileptic disorders. The clinical spectrum ranges from self-limited neonatal seizures to epileptic encephalopathy and delayed development. Mutations in *KCNQ2* could be either gain- or loss-of-function which require different therapeutic approaches. To better understand genotype–phenotype correlation, more reports of patients and their mutations with elucidated molecular mechanism are needed. We studied 104 patients with infantile-onset pharmacoresistant epilepsy who underwent exome or genome sequencing. Nine patients with neonatal-onset seizures from unrelated families were found to harbor pathogenic or likely pathogenic variants in the *KCNQ2* gene. The p.(N258K) was recently reported, and p. (G279D) has never been previously reported. Functional effect of p.(N258K) and p.(G279D) has never been previously studied. The cellular localization study demonstrated that the surface membrane expression of Kv7.2 carrying either variant was decreased. Whole-cell patch-clamp analyses revealed that both variants significantly impaired Kv7.2 M-current amplitude and density, conductance depolarizing shift in voltage dependence of activation, membrane resistance, and membrane time constant (*Tau*), indicating a loss-of-function in both the homotetrameric and heterotetrameric with Kv7.3 channels. In addition, both variants exerted dominant-negative effects in heterotetrameric with Kv7.3 channels. This study expands the mutational spectrum of KCNQ2- related epilepsy and their functional consequences provide insights into their pathomechanism.

## Introduction

Infantile-onset epilepsy represents one of the most challenging pediatric neurological conditions to diagnose and manage. It occurs in 0.8–1.2 per 1000 live births^1–3^ and over 300 genes including *KCNQ2* have been demonstrated to be involved in the pathogenesis of infantile-onset epilepsy^4^. Mutations in the *KCNQ2* gene can cause a wide range of severity ranging from benign/self-limited neonatal seizures (MIM#121200) to developmental and epileptic encephalopathy (DEE; MIM#613720)^4^. In general, patients with *KCNQ2* mutations have seizures that occur during the first week of life. In patients with benign/self-limited neonatal seizures, the seizures stops after weeks to months and the developmental trajectory is typically within normal range^5,6^. On the other hand, DEE phenotype is characterized by drug-resistant epilepsy and significant developmental delay^7–9^. Identifying causative mutations and its phenotypes is critical for genetic counseling and patient management.

The *KCNQ2* gene encodes Kv7.2 protein subunits that assemble the homotetrameric voltage-gated potassium channels (Kv7.2 channels). In addition, it can connect with Kv7.3, encoded by the *KCNQ3* gene, to form a heterotetrameric channels (Kv7.2/Kv7.3). Each subunit of Kv7.2 protein consists of intracellular amino (N) and carboxy (C) terminals, six transmembrane segments (S1–S6), and a pore loop between S5 and S6. Transmembrane segments between S1 and S4 forms a voltage sensing domain (VSD). The N-terminus (-NH) is tetramerization domain that determines the specificity of subunit assembly^10–15^. Kv7.2/Kv7.3 channels are mainly located in the soma and axon of neurons while Kv7.2 is expressed in axon terminals of neurons^16^. Both channels underlie the M-current, a slowly excitation current, non-inactivating current, a subthreshold voltage-gated potassium current that regulates neuronal excitability and is inhibited by muscarinic receptor agonists^17^.

To date, at least one hundred ninety-four cases of *KCNQ2*-related epilepsy have been reported with mutations distributed all over the gene leading to varying severity^18,19^. The functional study of the Kv7.2 channels may lead to a better understanding of the pathogenesis and disease severity, and may provide a guideline for treatments. Here, we report the clinical characteristics of nine patients with neonatal-onset seizures with *KCNQ2* mutations. Experiments to elucidate their electrophysiological functional effects of two variants were performed.

## Methodology

### Patients and clinical data collection

The institutional review board of the Faculty of Medicine, Chulalongkorn University approved this study (IRB No. 264/62) which follows the Declaration of Helsinki Guidelines and all subsequent amendments. Written informed consents were obtained from parents or legal guardians of the participants. From June 2016 to December 2020, we studied 104 patients with infantile-onset pharmaco-resistant epilepsy, defined as failure of adequate trials of two antiepileptic drugs^20^ who underwent exome or genome sequencing at King Chulalongkorn Memorial Hospital. The cohort is described in the authors’ previous work^21^. Nine patients were found to harbor pathogenic or likely pathogenic variants in the *KCNQ2* gene. The detailed demographic data and clinical characteristics were collected. The diagnosis of benign/self-limited neonatal epilepsy (OMIM#121200) or *KCNQ2*-related developmental and epileptic encephalopathy (DEE; OMIM#613720) weremade based on ILAE Classification and Definition of Epilepsy Syndromes with Onset in Neonates and Infants^22,22^.

### Exome, genome sequencing, bioinformatics and variant prioritization

Trio exome or genome was performed for all nine families. Genomic DNA was isolated from peripheral blood leucocytes. For exome sequencing, the DNA was enriched by SureSelect Human All Exon V5 kits (Agilent Technologies, Santa Clara, CA) and sent to Macrogen Inc., Seoul, South Korea. Illumina HiSeq 2000 Sequencer was used with a target output of 6 GB. For genome sequencing, DNA was sent to Beijing Genomics Institute (BGI), China. Sequence reads in FASTQ sequencing files were aligned to the Human Reference Genome hg19 from UCSC using Burrows-Wheeler Alignment (BWA) software (http://bio-bwa.sourceforge.net/). Single nucleotide variants (SNVs) and small insertions/ deletions (indels) were detected by GATK Haplotypecaller and annotated by dbSNP&1000G.

A list of 728 genes associated with Genetic Epilepsy Syndrome according to Genomics England PanelApp Version 2.2 (https://nhsgms-panelapp.genomicsengland.co.uk/panels/402/v2.2) were used for the first step of analysis. The analysis included gene list outside the “green entities" with high levels of evidence for disease association. In silico analysis including SIFT (http://sift.jcvi.org/); Polyphen-2, (http://genetics.bwh.harvard.edu/pph2/); M-CAP (http://bejerano.stanford.edu/mcap/); CADD (https://cadd.gs.washington.edu/); recommended pathogenicity threshold > 20) were also used to predict variants’ pathogenicity. Variants were considered novel if they were not previously reported in Genome Aggregation Database (gnomAD V3.1.2; https://gnomad.broadinstitute.org/), ClinVar (https://www.ncbi.nlm.nih.gov/clinvar/), not documented in PubMed scientific literature, and were not identified in our in-house Thai reference exome database (T-REx)^23^. Variants were classified according to the recommendation of American College of Medical Genetics and Genomics (ACMG)^24^.

### Plasmid construction, protein expression and localization in HEK293 cells

pcDNA3.1 + /KV7.2-DYK (#NM_004518.6) and pcDNA3.1 + /KV7.3-DYK (# NM_004519.4) were synthesized by Genscript (Piscataway, New Jersey, USA). cDNAKv7.2 was subcloned into the pcDNA3.1/CT-GFP-TOPO Vector (Invitrogen, Waltham, Massachusetts, USA), resulting in a C-terminal GFP, used to identify transfected cells. The two novel mutations, c.774C > G p.(N258K) and c.836G > A p.(G279D), were introduced using QuikChange site-directed mutagenesis kit (Agilent Technologies, Santa Clara, California, USA). Mutagenic primers are listed in Supplemental Table S1. All plasmids were verified using Sanger sequencing.

HEK 293 cells were transiently transfected with 2.5 μg of either wild-type, p.(N258K), or p.(G279D) Kv7.2 for homotetrameric experiments using Lipofectamine 3000 (Invitrogen). For heterotetrameric experiments, different types of Kv7.2 were co-transfected with wild-type Kv7.3 (wild-type Kv7.2/Kv7.3, p.(N258K) Kv7.2/Kv7.3, p.(G279D) Kv7.2/Kv7.3, wild-type Kv7.2/p.(N258K) Kv7.2/Kv7.3, wild-type Kv7.2/p.(G279D) Kv7.2/Kv7.3) at a 1:1 ratio (1.25g: 1.25g) or 1:1:2 ratio (0.625g: 0.625 g: 1.25 g) and assayed 48- or 72- hours post transfection.

Western blot analysis was used to determine Kv7.2 expressions in transfected HEK293 cells. Cells were scraped, centrifuged and resuspended. Membrane protein and cytoplasmic protein were extracted using the Mem-PER Plus Membrane Protein Extraction Kit (Thermo Fisher). The membrane protein and cytoplasmic protein were separated based on their hydrophobicity using two different buffers, a permeabilization buffer and a solubilization buffer. After that, 20 ug of denatured proteins were separated on 8% sodium dodecyl sulfate–polyacrylamide gel electrophoresis and transferred onto polyvinylidene difluoride membranes (Invitrogen). Transferred membranes were blocked for 1 h at room temperature (RT) in 5% nonfat dry milk and incubated overnight at 4 °C with monoclonal anti-Kv7.2 Rabbit mAb (1:500) (Cell Signaling Danvers, Massachusetts, USA) in 5% Bovine serum albumin (BSA) and then with **monoclonal anti-**GAPDH Rabbit (1:500) (Cell Signaling) in 5% BSA as a loading control. The membranes were then incubated for 2 h at RT with Anti-rabbit IgG, horseradish peroxidase (HRP)-linked Antibody (1:1000) (Cell Signaling) in 5% BSA. Specific protein bands were visualized using the ImageQuant Las4000 chemi-image (GE Healthcare).

For immunofluorescence, cells were fixed with 4% paraformaldehyde (PFA) for 15 min, permeabilized with 0.2% Triton X-100 for 20 min (heterotetrameric channels), and then blocked with 1% BSA for 1 h at RT. Cells were labeled overnight at 4 °C with polyclonal anti- KCNQ3 (1:800) (Invitrogen# PA1-930) in 0.1% BSA, then incubated with a Donkey Anti-Rabbit IgG H&L(1:500) (Alexa Fluor 647, abcam) in 0.1% BSA for 2 h at RT and mounted using ProLong Gold Antifade Mountant (Invitrogen# P36934). Confocal images were obtained using a Zeiss axio observer z1 microscope equipped with a 63 × oil immersion lens. The Zen 3.4 (blue edition) software was used for image analysis.

### Electrophysiological and potassium (K +) gating properties data acquisition and analysis

At 48-h post transfections, cells were sorted using fluorescence activated cell sorting (FACS) and seeded onto Poly-D-lysine (2 mg/mL) pre-coated 35 mm petri dishes. Potassium currents were investigated by standard whole-cell patch-clamp technique, using an Axopatch 200-B amplifier (Axon Instruments, Inc, Melbourne, Australia), controlled by a Digidata 1440A digitizer. The pCLAMP software (Version 10, Axon Instruments, Inc) was used for data acquisition and analysis. The extracellular solution contained (in mM) the following: 145 NaCl, 5 KCl, 2 CaCl_2_, 1 MgCl2, 10 glucose, and 10 HEPES, pH 7.3–7.4 titrated with NaOH; osmolality 315–320 mOsm/Kg. Microelectrodes (borosilicate glass capillaries BF150-86-10, Sutter/USA) had resistances of 1–3 MΩ when pulled with intracellular solution contained (in mM) the following: 140 K-gluconate, 2 MgCl_2_, 1 CaCl_2,_ 10 EGTA, 10 HEPES, and 10 Mg‐ATP, pH 7.3–7.4 titrated with KOH; osmolality 285–290 mOsm/Kg. Cells were held at –80 mV, voltage steps of 10 mV increment during 1.3 s from − 110 mV to + 50 mV and tail currents were recorded at a potential of − 40 mV.

Electrophysiological properties were demonstrated by representative raw M-current traces, current–voltage (I–V) curves were used to estimate the conductance and reversal potential, and M-current density was used to estimate electrical capacitance^16^. It was calculated as peak current (pA) at + 50 mV divided by the cell capacitance (pF). K + gating properties were investigated by the half-activating voltage (V_1/2_) detected depolarization of channels, the slope conductance (k) of channels. There were calculated by the Boltzmann function: I = 1/[1 + exp(V½–V)/k], V½ = half-activating voltage, k = slope, at tail current amplitudes at − 40 mV. The membrane resistance (Rm) was calculated according to Ohm’s law (V = IR), I is the current, V is the voltage and R is the resistance detected the resistance of the cell membrane when ions flow through it^21^. The membrane time constant *(Tau)* were calculated by Rm·Cm, Cm is membrane capacitance detected time of change membrane potential^25^.

### Data analysis

Data are expressed as means ± S.E.M. Statistical analyses were carried out using SPSS and GraphPad Prism 9.4.0. One-way ANOVA followed by Tukey’s post-hoc test was used to determine the statistical difference of experimental responses from untransfected cells and transfected cells, as well as the responses between wild type and mutants. The numbers of samples (n) have been indicated in the figure legends. Statistical significance was defined by *p ≤ 0.05, **p ≤ 0.01, ***p ≤ 0.001.

## Results

### Clinical characteristics

Of the 104 unrelated patients with infantile-onset pharmacoresistant epilepsy, De novo heterozygous pathogenic or likely pathogenic (P/LP) variants in *KCNQ2* were identified in 9% (9/104) and account for 20% (9/44) of the neonatal-onset cases. 67% (6/9) of the patients with *KCNQ2*-related epilepsy were female. All of the patients were born at term and had seizures onset during the neonatal period. None had family history of epilepsy or developmental delay. Neuroimaging data were available in 7 out of 9 patients. 71% (5/7) patients showed unremarkable neuroimaging. The EEG data were available in eight patients (88%; 8/9). The findings included multifocal epileptiform discharges (63%; 5/8) and burst suppression pattern (37%; 3/8). The number of antiepileptic drugs used before genetic testing ranged from 2 to 6. The clinical characteristics of the patients are summarized in Table 1. Clinical characteristics and molecular findings in 8 patients (patient 1–3 and 5–9) have been described in the authors’ previous work^21^.

**Table 1:** Clinical and molecular characteristics of nine patients with *KCNQ2*-related neonatal seizures.

ID	Sex	GA at birth	Family history	Seizure onset	Semiologies	EEG	Neuroimaging (age)	Antiepileptic drugs	Age at last visit	Outcome	NGS	Variant (s)	Reported (PMID)/ Novel	Inheritance (allele from)	ACMG variant classification
1	F	Term	Neg	4 days	Spasms	NA	NA	VGB, TPM, PB, LEV, VPA	1 Y and 8 Mo	Severe GDD	ES-trio	c.601C > T (p.R201C)	Reported (25740509)	AD *(*de novo*)*	Pathogenic
2	F	Term	Neg	2 days	GT, sequential	Multifocal IED	MRI: unremarkable	LEV Clonazepam	9 Mo	Severe GDD	ES-trio	c.601C > T (p.R201C)	Reported (25740509)	AD *(*de novo*)*	Pathogenic
3	M	Term	Neg	3 days	Myoclonus, eyes deviate left, head turn left	Multifocal IED	MRI: unremarkable (9 days)	LEV, PB	1 Y and 6 Mo	Normal milestones	ES-trio	c.774C > G p.(N258K) *	Reported (35468861)	*AD (*de novo*)*	Pathogenic
4	F	Term (twin A; unaffected twin B)	Neg	2 days	GT	Suppression-burst	MRI: cerebral atrophy, corpus callosum agenesis (7 Mo)	PHT, PB, TPM, LEV, VGB, perampanel	2 Y and 9 Mo	Severe GDD	GS-trio	c.821C > T (p.T274M)	Reported (24318194)	AD *(*de novo*)*	Pathogenic
5	F	Term	Neg	20 h	GT, Multifocal clonic	Suppression-burst	CT: unremarkable	PB, LEV, TPM, perampanel	1 Y and 7 Mo	Severe GDD	ES-trio	c.821C > T, (p.T274M)	Reported (24318194)	AD *(*de novo*)*	Pathogenic
6	F	Term	Neg	1 day	GT, sequential	Suppression-burst	MRI: unremarkable (1 Mo)	PB, LEV, TPM, PHT, Clonazepam, perampanel	3 Y and 5 Mo	Severe GDD	ES-trio	c.836G > A p.(G279D) *	Novel	AD *(*de novo*)*	Likely pathogenic
7	M	Term	Neg	36 h	GT	Multifocal IED	NA	LEV, PB	1 month	NA	ES-trio	c.881C > T (p.A294V)	Reported (26138355)	*AD (*de novo*)*	Pathogenic
8	F	Term	Neg	10 days	GT	Multifocal IED	MRI: cerebral atrophy (11 Mo)	PB, LEV, TPM, diazepam	3 Y and 8 Mo	Severe GDD	ES-trio	c.1657C > T (p.R553W)	Reported (32117026)	AD *(*de novo*)*	Pathogenic
9	M	Term	Neg	19 h	GT	Multifocal IED	MRI: unremarkable (2 weeks)	PB, TPM, LEV, CBZ	4 Mo	Brief eye contact/not fix and follow, head lag	ES-trio	c.1687G > A (p.D563N)	Reported (30669290)	AD *(*de novo*)*	Pathogenic

CBZ, carbamazepine; CT, computerized tomography; EEG, electroencephalogram; F, female; FLAIR, fluid attenuation inversion recovery; GDD, global developmental delay; GT, generalized tonic seizures; GTC, generalized tonic clonic seizures; rs, hours; ID, identification; IED, interictal epileptiform discharges; LEV, Levetiracetam; LGA, large for gestational age M, male; Mo, months; MRI, magnetic resonance imaging; NA, not available; NCSE, nonconvulsive status epilepticus; Neg, negative; NGS, next-generation sequencing; P5P, pyridoxal-5-phosphate; PB, phenobarbital; PHT, phenytoin; SE, status epilepticus; TPM, topiramate; VGB, vigabatrin; VPA, valproate; ES, whole-exome sequencing; GS, whole-genome sequencing; Y, year.

*The variants’ functional effects were studied.

### Molecular characteristics

**Figure 1:**
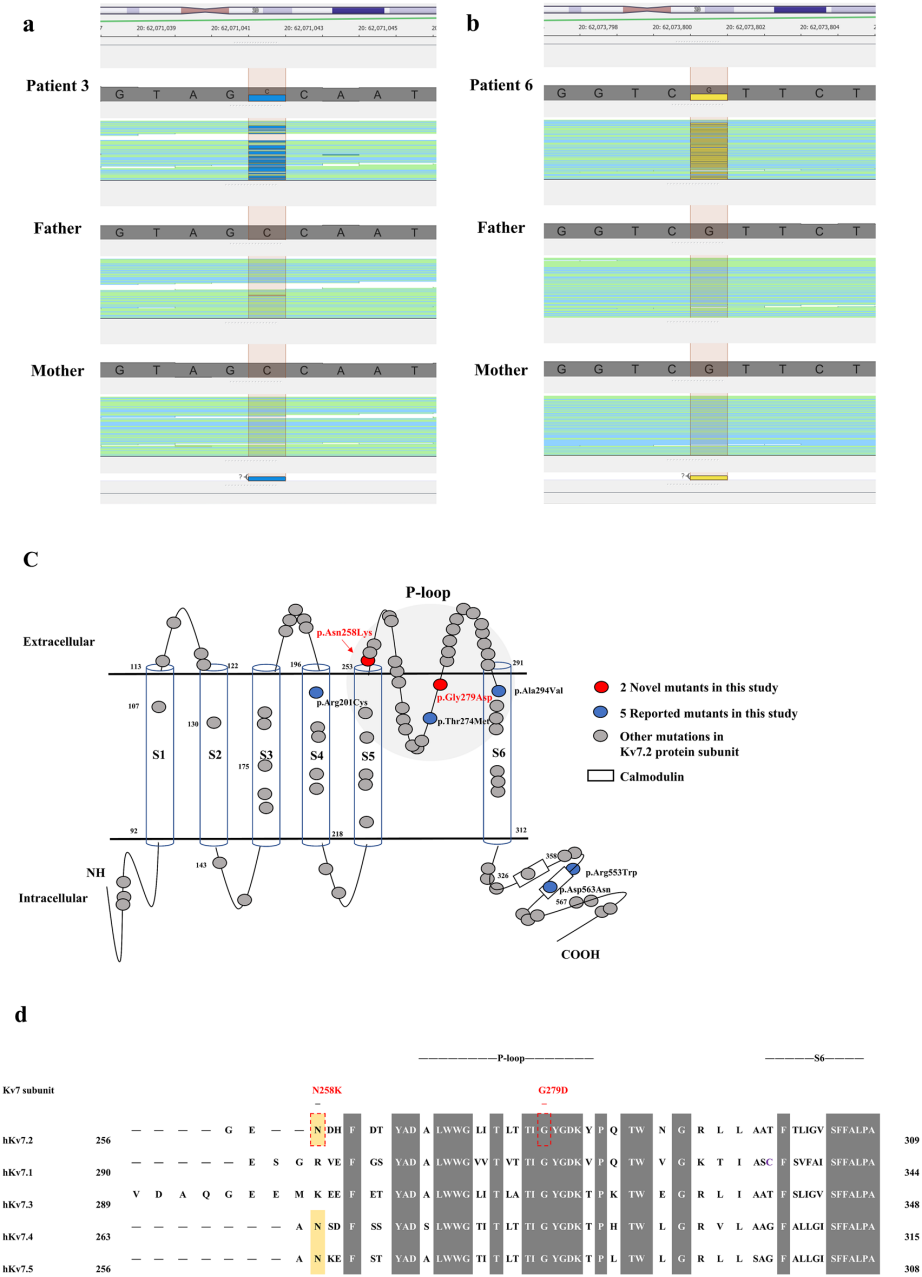
Sequence alignment and schemes representation of Kv7 subunits with different mutation locations in Kv7.2 subunits. (**a**–**b**) The patients’ BAM (Binary alignment mapping) capture on Golden Helix Genome Browse 3.0.0 which position was de novo missense mutations in reverse strand. The total yield of read depth was 104/85 (Reference/Alternate) for the p.(N258K) variant and 72/33 (Reference/Alternate) for the p.(G279D) variant. (**c**) Predicted position of missense mutations in the S5-H5 linker and pore loop domain (p.(N258K), p.(G279D)). (**d**) Alignment of human Kv7 subunits and location of amino acids mutated in the pore loop domain to the beginning of S6 among Kv7.2 subunits. Grey boxes are highly conserved sequences of Kv7 subunits. Yellow boxes are critical sequences of the voltage-gated K + channel subunits.

Seven de novo missense P/LP variants were found in nine patients (Table 1). The p.(N258K) was recently reported during the course of this study. The p.(G279D) variants have never been previously reported. (Fig. 1a,b). In silico analysis predicted the variants to be disease-causing (Supplementary Table S2). The Asparagine and glycine residue are located in the pore-loop domain of the K_V_7.2. The G279D variant is highly conserved across members of the Kv7 family whereas the N258K is located in a poorly conserved region at the junction of S5/re-entrant loop (Fig. 1c,d).

### p.(N258K) and p.(G279D) impairs cell surface expression of Kv7.2

**Figure 2:**
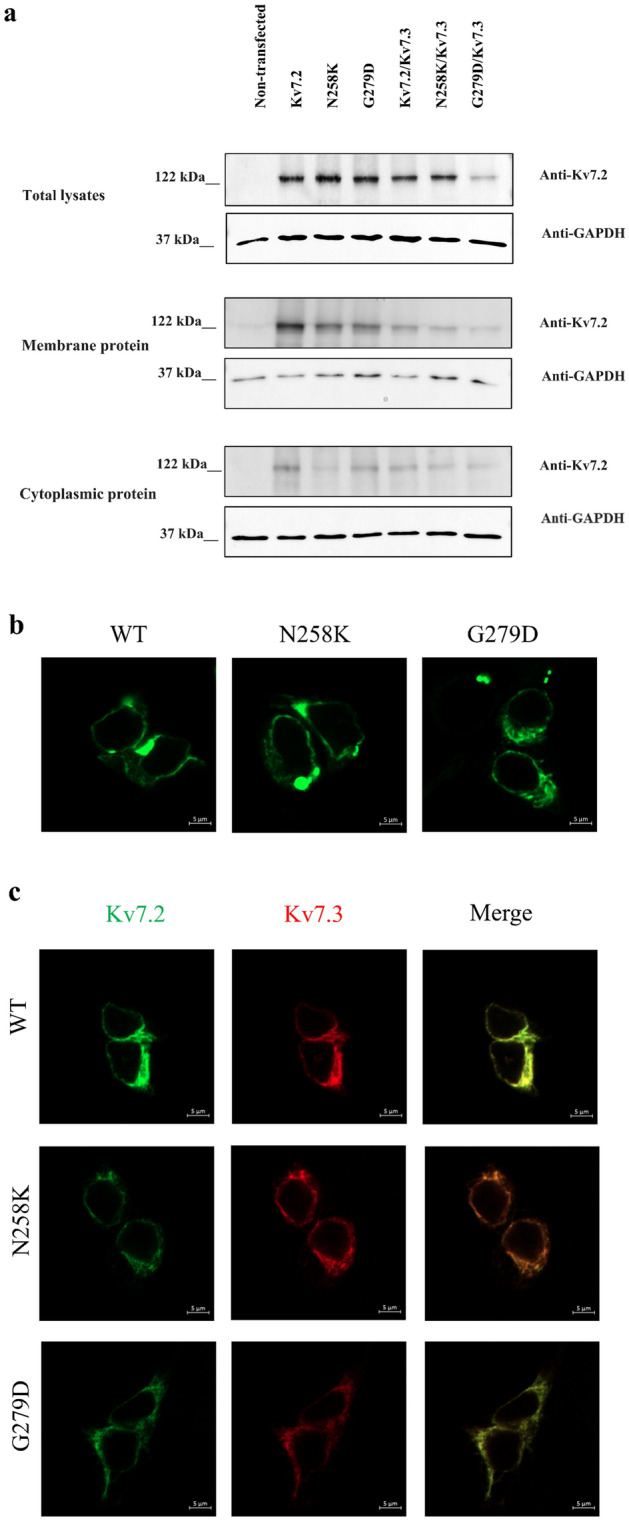
p.(N258K) and p.(G279D) Kv 7.2 diminishes Kv 7.2 surface membrane expression. (**a**) Western blots analysis of Kv 7.2 protein expressions in membrane, cytoplasmic and total cell lysate of cells expressing Kv7.2WT, p.(N258K) and p.(G279D), WT Kv7.2/Kv7.3, or p.(N258K) or p.(G279D) co-expressed with Kv7.3. Non- transfected HEK293 cells was used as a negative control and GAPDH as a loading control. (**b**) Representative confocal microscopy images of HEK 293 cells expressing either Kv7.2WT, p.(N258K) or p.G27D. Kv7.2WT (left panel), p.(N258K) (middle panel) and G279D (right panel). Scale bar: 5 µm. (**c**) Representative confocal microscopy images of cells expressing Kv7.2WT, p.(N258K) and p.(G279D), WT Kv7.2/Kv7.3, or p.(N258K) or p.(G279D) co-expressed with Kv7.3. Scale bar: 5 µm.

To understand the functional impact of p.(N258K) and p.(G279D) Kv7.2, we first assessed Kv7.2 cellular localization in cells expressing homomeric and heteromeric channels. In comparison to wild-type (WT) channels, both of p.(N258K) and p.(G279D) Kv7.2 exhibited lower levels of membrane expression seen in both Western blot (Fig. 2a) and immunofluorescence (Fig. 2b,c).

### Electrophysiological functions of novel mutations forming homotetrameric channels

**Figure 3:**
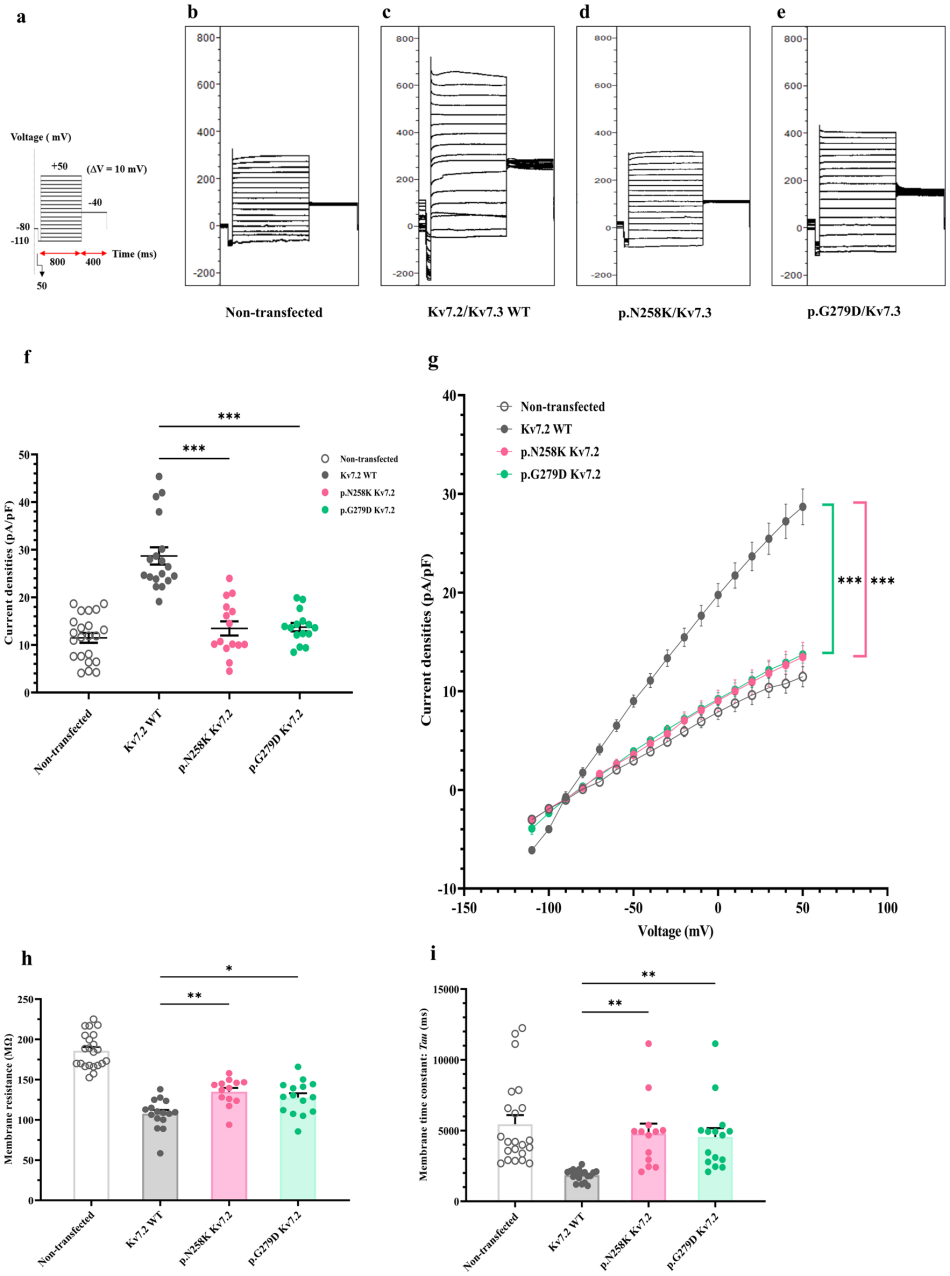
Whole cell patch clamp recordings of K + currents in of homotetrameric Kv7.2. K + currents recorded in non-transfected cells or cells expressing WT Kv7.2, p.(N258K) or p.(G279D) Kv 7.2. (**a**) Voltage protocol with voltage-clamp steps were from -110 mV to + 50 mV in 10 mV increments. (**b**–**e**s) Representative current traces of non-transfected (n = 22) (**b**), or cells expressing Kv7.2WT (n = 18) (**c**), p.(N258K) (n = 15) (d), and p.(G279D) (n = 15) (**e**). (**f**) Average peak M-current densities (pA/pF) at + 50 mV. (**g**) Conductance–voltage (I-V) relationships, significant differences from − 50 to + 50 mV and reversal potential. (**h**) membrane resistance (Rm). (i) membrane time constant *(Tau)*. One-way ANOVA followed by Tukey post-hoc test, *p < 0.05, **p < 0.01, ***p < 0.001.

To analyze the electrophysiological functions of p.(N258K) and p.(G279D) Kv7.2, whole-cell patch clamp was performed using voltage-clamp steps from − 110 mV to + 50 mV in 10 mV increments (Fig. 3a). Cells expressing p.(N258K) and p.(G279D) Kv7.2 exhibited lower M-current amplitude and current density when compared to WT Kv7.2 (Fig. 3b–f). The conductance-voltage relationship shifted significantly towards depolarized voltages for p.(N258K) and p.(G279D) Kv7.2 compared to WT Kv 7.2, resulting in a more positive half-activating voltages (V_1/2_) and lower slope conductance (K) (Fig. 3g, Supplementary Table S3). Input resistance and time constant (Tau) in cells expressing p.(N258K) and p.(G279D) Kv7.2 were significantly higher than in WT cells (Fig. 3h, i, Supplementary Table S3).

### Electrophysiological functions of novel mutations in heterotetrameric channels

**Figure 4:**
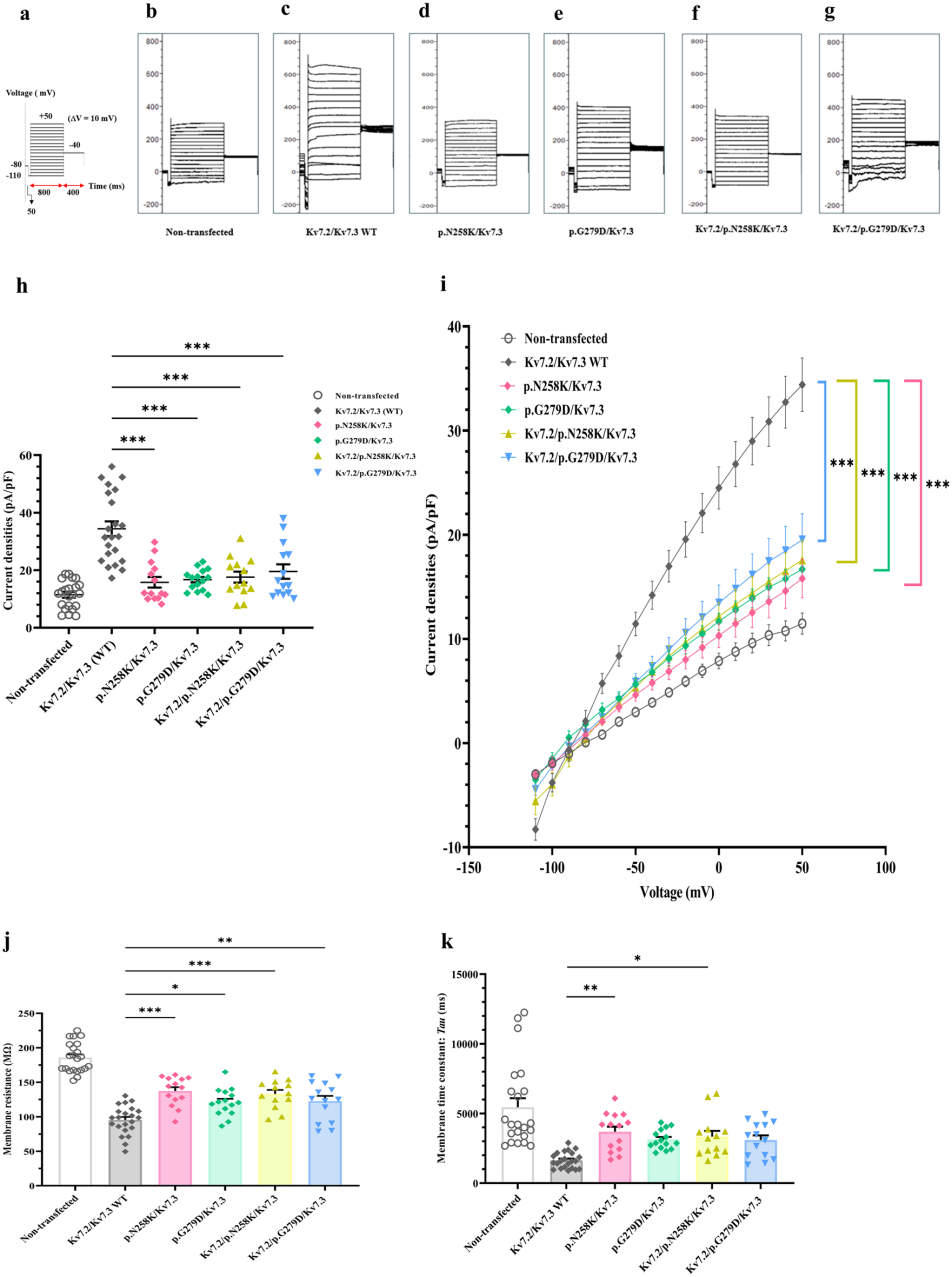
Whole cell patch clamp recordings of K + currents in of heterotetrameric Kv7.2/Kv7.3 channels. K + currents recorded in non-transfected cells or cells expressing Kv7.2/7.3 WT, p.(N258K) or p.(G279D) co-expressed with WT Kv 7.3 or WT Kv 7.2/7.3 (**a**) Voltage protocol with voltage-clamp steps as Fig. 3a. (**b**–**g**) Representative raw current traces for non-transfected cells (n = 22) **(b)**, Kv7.2/Kv7.3WT (n = 22) (**c**), p.(N258K)/Kv7.3 (n = 14) (**d**), p.(G279D)/Kv7.3(n = 15) (e), Kv7.2/p.(N258K) /Kv7.3 (n = 13) (f), Kv7.2/G279D/Kv7.3(n = 14) (g). (**h**) average peak M-current densities (pA/pF) at + 50 mV. **(i)** Conductance–voltage (I–V) curves, significant differences from − 50 to + 50 mV. (**j**) membrane resistance (Rm). (**k**) membrane time constant *(Tau).* One-way ANOVA followed by Tukey post-hoc test, *p < 0.05, **p < 0.01, ***p < 0.001 based on one-way ANOVA Tukey test.

Given the heterozygous nature of the *KCNQ2*-associated epileptic disorders, we proceeded to evaluate the electrophysical functions and properties using voltage protocol steps as in homotetrameric channels (Fig. 4a) of cells expressing p.(N258K) or p.(G279D) Kv7.2 with Kv.7.3 to mimic heterozygous heterotetrametric channels or with WT Kv 7.2/Kv 7.3 to mimic homozygous heterotetrametric channels, aiming to disclose potential alterations introduced by the either p.(N258K) or p.(G279D) Kv7.2. Co-expressions of either p.(N258K) or p.(G279D) Kv7.2 with Kv7.3 at a 1:1 ratio, and p.(N258K) or p.(G279D) Kv7.2 with Kv7.2/Kv.7.3 at a 1:1:2 ratio was compared to a 1:1 co-expression of Kv7.2/Kv7.3 WT channels. The M-current amplitude and current density in the co-expressions were significantly lower than in WT channels (Fig. 4b–h). Conductance-voltage relationships were significantly shifted towards depolarization voltages, yielding a more positive V_1/2_ for co-expressions compared to WT channels (Fig. 4i and Supplementary Table S3). Membrane resistance for co-expressions were significantly higher than WT channels. For time constants, only p.(N258K) Kv7.2 co-expressed as a heterotetrametric or homozygous heterotetrametric constellations were significantly higher than WT channels, whilst p.(G279D) Kv 7.2, though produced higher time constants, did not differ significantly (Fig. 4j,k). Together, this demonstrates that p.(N258K) and p.(G279D) Kv7.2 exhibit strong dominant negative effects on WT Kv 7.2 in both heterozygous heterotetrametric or homozygous heterotetrametric constellations.

## Discussion and conclusions

This study reports the clinical and molecular characteristics of nine Thai patients with *KCNQ2*-related epilepsy. The observed phenotypes of KCNQ2-related disorders in this study are benign/self-limited neonatal seizures and developmental and epileptic encephalopathy. The phenotypic spectrum of the patients in this cohort are similar to previously reported cases.

This study reports one novel p.(G279D) variant. The p.(N258K) variant has been recently reported in a neurodevelopmental disorder cohort^26^, though the functional effects of p.(N258K) has never been previously studied. Benign/self-limited neonatal seizures phenotype is associated with truncated heterozygous loss-of-function mutations, and DEE is usually associated with de novo missense mutations^4,16,27^. While benign/self-limited neonatal seizures-associated variants are distributed throughout the Kv7.2 protein, mutations associated with KCNQ2-related DEE usually located in the voltage sensor domain, the pore, the C-terminus proximal region, and the calmodulin-binding B helix region^4,19,27,28^. However, there are previous case reports mutations in the pore domain that display variable phenotypes including benign/self-limited neonatal seizures and DEE^29^.

Both the p.(N258K) and p.(G279D) mutation are in the pore-domain. The p.(G279D) has never been previously reported. The previously reported cases with different changes at G279 residue including G279S, G279V and G279C show DEE phenotype. The p.(N258K) variant was found in a neurodevelopmental disorder patient cohort^26^. The epilepsy phenotype for p.(N258K) in the previous report is unspecified. Yalcin et al. and Maljevic et al. reported benign/self-limited familial neonatal epilepsy patients with p.N258S mutation which is at the same amino acid position^30^. A patient with a de novo p.N258S mutation mentioned in another study by Lee J et al. has neonatal seizures with moderate developmental delay^31^. This suggests that other factors including environmental and perinatal events, epigenetic, genetic modifiers can potentially influence the variability of the phenotypes.

Different amino acid changes at the same amino acid residues may not have the same molecular pathomechanism. For example, the functional study of p.R213Q and p.R213W mutations in *KCNQ2* have showed that p.R213Q lead to more severe functional changes than p.R213W. Consistently, the p.R213Q caused neonatal-onset epileptic encephalopathy (EE), while the p.R213W caused benign familial neonatal convulsions^28^.

Different molecular pathomechanism leads to dissimilar disease severity and natural courses. Truncated *KCNQ2* variants predicted to cause haploinsufficiency are associated with self-limited neonatal epilepsy, while phenotypic spectrum of missense variants ranges from self-limited neonatal epilepsy to severe developmental and epileptic encephalopathy (DEE) phenotype. *KCNQ2* pathogenic variants in patients with developmental delay without seizures or with seizures outside the neonatal period exhibit gain of channel function when expressed in vitro^27,32^. Therefore, functional studies are needed for better understanding of genotype–phenotype correlation.

The variant, p.(N258K), found in Patient 3 is in the S5-H5 linker. The asparagine at amino acid residue 258 is poorly conserved sequence among members of the voltage-gated K + channels. It is located towards the end of the pore or through the extracellular part of the pore which may have conformational change during the depolarization and affect pore conductance and opening of the channel^30^. This is supported by p.N258S mutation which was previously shown to lead to a rearrangement of the pore region^33^. The patch-clamp experiments confirmed the pathogenicity of the p.(N258K) (Supplementary Table. S3), showing both loss-of-function and dominant negative effects. Both effects were previously demonstrated in some mutations in this gene including p.A265P^28^.

The variant, p.(G279D), found in Patient 6 is in the pore-forming S5 and S6 regions. Glycine 279 is highly conserved in pore domain of the Kv7 channel family^34,35^. Additionally, the Gly279 position is a part of the GYG pore motif which is a signature sequence of the selectivity filter (K +) and channel conductance and stability of the open pore state^36,37^. Mutations in this location often lead to abnormal selectivity filter (K +). The findings of the patch-clamp corroborate the pathogenicity of p.(G279D) in being loss-of-function and dominant negative. The previously reported p.G279S mutation also had a loss-of-function and dominantly negative effect^38^.

In our experimental model, the reduction of M-current, M-current densities, conductance and increment of depolarizing voltage, membrane resistance (Rm) observed for the p.(N258K) and p.(G279D) variants did not differ. A caveat is that all experiments were performed at RT; therefore the results may not be identical to physiological temperature.

Interestingly, the membrane time constant (*tau*) of p.(N258K) was increased in both the homotetramic and heterotetramic channels. Because of the more membrane capacity (more swollen cells), channels may need more time to change the membrane potential to return to the "resting state". Conversely, the clinical outcome in the Patients 3 and 6 differs greatly. Patient 3 has neonatal-onset seizures. The seizures were controlled after two anti-seizures medications. with normal developmental milestones at age one year and six months. While Patient 6 has DEE phenotype. This suggests the complexity of correlating in vitro missense variant functional effects and phenotype. Additional genetic modifiers, epigenetic factors and the extent of derangement of channel function/localization may be important contributors of disease severity.

In conclusion, two novel variants, p.(N258K) and p.(G279D), in the pore loop domain of Kv7.2 were identified and found to have loss-of-function and dominant-negative effects, expanding the mutational spectrum of the *KCNQ2*.

## Supplementary Information

Supplementary Information.

## Data Availability

The datasets used and/or analyzed during the current study are available from the corresponding author upon reasonable request. The molecular findings presented in this study have been deposited to ClinVar (https://www.ncbi.nlm.nih.gov/clinvar/) under accession numbers SCV002564399—SCV002564405.

## References

[1] Symonds JD (2019). Incidence and phenotypes of childhood-onset genetic epilepsies: A prospective population-based national cohort. Brain.

[2] Gaily E, Lommi M, Lapatto R, Lehesjoki AE (2016). Incidence and outcome of epilepsy syndromes with onset in the first year of life: A retrospective population-based study. Epilepsia.

[3] Stodberg T (2020). Epilepsy syndromes, etiologies, and the use of next-generation sequencing in epilepsy presenting in the first 2 years of life: A population-based study. Epilepsia.

[4] Goto A (2019). Characteristics of KCNQ2 variants causing either benign neonatal epilepsy or developmental and epileptic encephalopathy. Epilepsia.

[5] Ronen GM, Rosales TO, Connolly M, Anderson VE, Leppert M (1993). Seizure characteristics in chromosome 20 benign familial neonatal convulsions. Neurology.

[6] Singh NA (2003). KCNQ2 and KCNQ3 potassium channel genes in benign familial neonatal convulsions: Expansion of the functional and mutation spectrum. Brain.

[7] Orsini A (2021). The role of inflammatory mediators in epilepsy: Focus on developmental and epileptic encephalopathies and therapeutic implications. Epilepsy Res..

[8] Nariai H, Duberstein S, Shinnar S (2018). Treatment of epileptic encephalopathies: Current state of the art. J. Child Neurol..

[9] Kalser J, Cross JH (2018). The epileptic encephalopathy jungle - from Dr West to the concepts of aetiology-related and developmental encephalopathies. Curr. Opin. Neurol..

[10] Biervert C (1998). A potassium channel mutation in neonatal human epilepsy. Science.

[11] Cooper EC, Harrington E, Jan YN, Jan LY (2001). M channel KCNQ2 subunits are localized to key sites for control of neuronal network oscillations and synchronization in mouse brain. J. Neurosci..

[12] Lerche H (1999). A reduced K+ current due to a novel mutation in KCNQ2 causes neonatal convulsions. Ann. Neurol..

[13] Soldovieri MV (2016). Early-onset epileptic encephalopathy caused by a reduced sensitivity of Kv7.2 potassium channels to phosphatidylinositol 4,5-bisphosphate. Sci. Rep..

[14] Xu M, Cooper EC (2015). An Ankyrin-G N-terminal gate and protein kinase CK2 dually regulate binding of voltage-gated sodium and KCNQ2/3 potassium channels. J. Biol. Chem..

[15] Zaydman MA, Cui J (2014). PIP2 regulation of KCNQ channels: Biophysical and molecular mechanisms for lipid modulation of voltage-dependent gating. Front. Physiol..

[16] Dirkx N, Miceli F, Taglialatela M, Weckhuysen S (2020). The role of Kv7.2 in neurodevelopment: Insights and gaps in our understanding. Front. Physiol..

[17] Brown DA, Adams PR (1980). Muscarinic suppression of a novel voltage-sensitive K+ current in a vertebrate neurone. Nature.

[18] Zhang J (2020). Identifying mutation hotspots reveals pathogenetic mechanisms of KCNQ2 epileptic encephalopathy. Sci. Rep..

[19] Millichap JJ (2016). KCNQ2 encephalopathy: Features, mutational hot spots, and ezogabine treatment of 11 patients. Neurol. Genet..

[20] Kwan P (2010). Definition of drug resistant epilepsy: Consensus proposal by the ad hoc Task Force of the ILAE Commission on Therapeutic Strategies. Epilepsia.

[21] Boonsimma P (2022). Exome sequencing as first-tier genetic testing in infantile-onset pharmacoresistant epilepsy: Diagnostic yield and treatment impact. Eur. J. Hum. Genet..

[22] Zuberi SM (2022). ILAE classification and definition of epilepsy syndromes with onset in neonates and infants: Position statement by the ILAE task force on nosology and definitions. Epilepsia.

[23] Shotelersuk V (2021). The Thai reference exome (T-REx) variant database. Clin. Genet..

[24] Richards S (2015). Standards and guidelines for the interpretation of sequence variants: A joint consensus recommendation of the American College of Medical Genetics and Genomics and the Association for Molecular Pathology. Genet. Med..

[25] Isokawa M (1997). Membrane time constant as a tool to assess cell degeneration. Brain Res. Brain. Res. Protoc..

[26] Hamanaka K (2022). Large-scale discovery of novel neurodevelopmental disorder-related genes through a unified analysis of single-nucleotide and copy number variants. Genome Med..

[27] Miceli F (2013). Genotype-phenotype correlations in neonatal epilepsies caused by mutations in the voltage sensor of K(v)7.2 potassium channel subunits. Proc. Natl. Acad. Sci. U S A.

[28] Orhan G (2014). Dominant-negative effects of KCNQ2 mutations are associated with epileptic encephalopathy. Ann. Neurol..

[29] Laccetta G (2019). A de novo KCNQ2 gene mutation associated with non-familial early onset seizures: Case report and revision of literature data. Front. Pediatr..

[30] Yalçin O (2007). A novel missense mutation (N258S) in the KCNQ2 gene in a Turkish family afflicted with benign familial neonatal convulsions (BFNC). Turk. J. Pediatr..

[31] Lee J, Lee C, Ki C-S, Lee J (2020). Determining the best candidates for next-generation sequencing-based gene panel for evaluation of early-onset epilepsy. Mol. Genet. Genom. Med..

[32] Miceli F (2022). KCNQ2 R144 variants cause neurodevelopmental disability with language impairment and autistic features without neonatal seizures through a gain-of-function mechanism. EBioMedicine.

[33] Maljevic S (2011). Temperature and pharmacological rescue of a folding-defective, dominant-negative KV 7.2 mutation associated with neonatal seizures. Hum. Mutat..

[34] Schroeder BC, Hechenberger M, Weinreich F, Kubisch C, Jentsch TJ (2000). KCNQ5, a novel potassium channel broadly expressed in brain, mediates M-type currents. J. Biol. Chem..

[35] McCoy JG, Nimigean CM (1818). Structural correlates of selectivity and inactivation in potassium channels. Biochim. Biophys. Acta.

[36] Chapman ML, Krovetz HS, VanDongen AM (2001). GYGD pore motifs in neighbouring potassium channel subunits interact to determine ion selectivity. J. Physiol..

[37] So I, Ashmole I, Davies NW, Sutcliffe MJ, Stanfield PR (2001). The K+ channel signature sequence of murine Kir2.1: mutations that affect microscopic gating but not ionic selectivity. J. Physiol..

[38] Peters HC, Hu H, Pongs O, Storm JF, Isbrandt D (2005). Conditional transgenic suppression of M channels in mouse brain reveals functions in neuronal excitability, resonance and behavior. Nat. Neurosci..

