# Supplementary material for: Nine patients with KCNQ2-related neonatal seizures and functional studies of two missense variants
Source: Sci Rep. 2023 Feb 27;13:3328. doi: 10.1038/s41598-023-29924-y (PMC9971330; doi:10.1038/s41598-023-29924-y)
Supplement: Supplementary file 1 — Supplementary Information. [file 41598_2023_29924_MOESM1_ESM.docx]

**SUPPLEMENTARY INFORMATION**

**TITLE:** **Nine patients with *KCNQ2*-related neonatal seizures and functional studies of two missense variants**

**AUTHORS:** Chokvithaya S^1,2,3^, Caengprasath N^1,2^, Buasong A^1,2^, Jantasuwan S^1,2^, Santawong K^1,2^, Leela-adisorn N^7^, Tongkobpetch S^1,2^, Ittiwut C ^1,2^, Saengow V^6^, Kamolvisit W^1,2^, Boonsimma P^1,2*^, Bongsebandhu-Phubhakdi S^4,5^^*^, Shotelersuk V^1,2^

**Author details:**

1. Center of Excellence for Medical Genomics, Medical Genomics Cluster, Department of Pediatrics, Faculty of Medicine, Chulalongkorn University, Bangkok 10330, Thailand
2. Excellence Center for Genomics and Precision Medicine, King Chulalongkorn Memorial Hospital, The Thai Red Cross Society, Bangkok 10330, Thailand, Department of The Excellence Center for Genomics and Precision Medicine, Faculty of Medicine, Chulalongkorn University, Bangkok, Thailand
3. Department of Clinical Pathology and Medical Technology Laboratory, Queen Sirikit National Institute of Child Health, Ministry of Public Health, Bangkok, Thailand.
4. Department of Physiology, Faculty of Medicine, Chulalongkorn University, Bangkok, Thailand.
5. Chula Neuroscience Center, King Chulalongkorn Memorial Hospital, The Thai Red Cross Society, Bangkok, Thailand.
6. Department of Pediatrics, Maharat Nakhon Ratchasima Hospital, Nakhon Ratchasima, Thailand.
7. Department of Stem Cell and Cell Therapy Research Unit, Faculty of Medicine, Chulalongkorn University, Bangkok, Thailand

**Corresponding Author:**

* Saknan Bongsebandhu-Phubhakdi, Ph.D. Department of Physiology, Faculty of Medicine, Chulalongkorn University, Bangkok 10330, Thailand. Tel: 662-256-4267; E-mail: saknan@live.jp, saknan.b@chula.ac.th

* Ponghatai Boonsimma, M.D, Center of Excellence for Medical Genomics, Medical Genomics Cluster, Department of Pediatrics, Faculty of Medicine, Chulalongkorn University, Bangkok 10330, Thailand.Tel: 662-256-3354; E-mail: [**Ponghatai.B@chula.ac.th**](mailto:Ponghatai.B@chula.ac.th)

**Supplementary Table S1. List of primer sequences used for amplified the *KCNQ2* gene (a) and Quick-Change site-directed mutagenesis (b).**

| **a** |  |  |
| --- | --- | --- |
| **Fragment** | **Forward Primer (5'-3')** | **Reverse Primer (5'-3')** |
| ***KCNQ2* gene** | AAAAGCTTCCAGGCACCATGGTGCAG | TCTTCCTGGGCCCGGCCCAG |
|  |  |  |
| **b** |  |  |
| **Fragment** | **Forward Primer (5'-3')** | **Reverse Primer (5'-3')** |
| **c.774C>G p.(N258K)** | GAAGGGGGAGAAGGACCACTTTGACACCTA | TAGGTGTCAAAGTGGTCCTTCTCCCCCTTC |
| **c.836G>A p.(G279D)** | TGACCACCATTGACTACGGGGACAAGTACC | GGTACTTGTCCCCGTAGTCAATGGTGGTCA |

**Supplementary Table S2. Interpretation of the identified variants in the *KCNQ2* gene**

|  | **Patient 1,2** | **Patient 3** | **Patient 4,5** | **Patient 6** | **Patient 7** | **Patient 8** | **Patient 9** |
| --- | --- | --- | --- | --- | --- | --- | --- |
| **Variant**† | c.601C>T  (p.R201C) | c.774C>G  p.(N258K) | c c.821C>T (p.T274M) | c.836G>A  p.(G279D) | c.881C>T  (p.A294V) | c.1657C>T  (p.R553W) | c.1687G>A  (p.D563N) |
| **Inheritance** | *De novo* | *De novo* | *De novo* | *De novo* | *De novo* | *De novo* | *De novo* |
| **SIFT** | D | D | D | D | D | D | D |
| **Polyphen-2** | P.D. | P.D. | P.D. | P.D. | P.D. | P.D. | P.D. |
| **M-CAP** | P.P. | P.P. | P.P. | P.P. | P.P. | P.P. | P.P. |
| **CADD** | 33 | 27.4 | 28.7 | 23.6 | 33 | 26.2 | 27.4 |
| **gnomAD** | - | - | - | - | - | - | - |
| **dbSNP** | rs727503974 | rs770187706 | rs796052623 | - | - | rs759584387 | rs796052653 |
| **Evidence of**‡ **pathogenicity** | PS1, PS2, PM2 | PS2, PM1, PM2, PM5, PP2 | PS2, PS3, PM2 | PS2, PM2, PP3 | PS2, PM1, PM2, PP3, PP5 | PS2, PM1, PM2, PP3, PP5 | PS2, PM1, PM2, PP3, PP5 |
| **Classification**‡ | Pathogenic | Pathogenic | Pathogenic | Likely pathogenic | Pathogenic | Pathogenic | Pathogenic |

*D, deleterious; P.D., probably damaging; P.P., possibly pathogenic*

SIFT, sorting intolerant from tolerant (http://sift.jcvi.org/); Polyphen-2, prediction of functional effects of human SNPs (http://genetics.bwh.harvard.edu/pph2/); M-CAP, Mendelian clinically applicable pathogenicity score (http://bejerano.stanford.edu/mcap/); CADD, combined annotation dependent depletion (https://cadd.gs.washington.edu/; recommended pathogenicity threshold >20); dbSNP (https://www.ncbi.nlm.nih.gov/projects/SNP/); gnomAD, https://gnomad.broadinstitute.org/

† NCBI (National Center of Biotechnology Information) reference sequence: NM_172107.4

‡ According to the American College of Medical Genetics and Genomics interpretations guidelines (PMID 25741868)

**Supplementary** **Table S3. Electrophysiological parameter of currents recorded in HEK293 cells transfected with the indicated plasmid**

**combinations**

| a |  |  |  |  |  |
| --- | --- | --- | --- | --- | --- |
|  | cDNA transfected (µg) | n | M-current at +50 mV (pA) | M-current density  (pA/pF at +50 mV) | % reduction  of M-current density |
|  |  |  |  |  |  |
| Non-transfected | 0 | 22 | 272.35 ± 6.60 | 11,47 ± 1.02 |  |
| Kv7.2 WT | 2.5 | 18 | 498.71 ± 29.27 | 28.02 ± 1.81 | 100 |
| p.(N258K) Kv7.2 | 2.5 | 15 | 374.52 ± 13.83 | ***13.46 ± 1.48 | 51.97 |
| p.(G279D) Kv7.2 | 2.5 | 15 | 402.33 ± 18.65 | ***14.19 ± 1.03 | 49.36 |
| Kv7.2/Kv7.3WT | 1.25:1.25 | 22 | 554.27 ± 31.89 | 34.40 ± 4.23 | 100 |
| p.(N258K)/Kv7.3 | 1.25:1.25 | 14 | 373.34 ± 17.64 | ***15.79 ± 1.83 | 54.09 |
| p.(G279D)/Kv7.3 | 1.25:1.25 | 15 | 424.10 ± 18.36 | ***16.69 ± 0.94 | 51.49 |
| Kv7.2/p.(N258K)/Kv7.3 | 0.625:0.625:1.25 | 13 | 385.26 ± 18.44 | ***17.58 ± 1.89 | 48.89 |
| Kv7.2/p.(G279D)/Kv7.3 | 0.625:0.625:1.25 | 14 | 431.91± 30.52 | ***19.52 ± 2.51 | 43.27 |

| b |  |  |  |  |  |  |  |
| --- | --- | --- | --- | --- | --- | --- | --- |
|  | cDNA transfected (µg) | n | V_1/2_ (mV)  at -40 mV | k (mV/efold) | Membrane resistance (MW) | Membrane time constant (*Tau*) (ms) | Reversal potential  (mV) |
|  |  |  |  |  |  |  |  |
| Non-transfected | 0 | 22 | -42.16 ± 0.0016 | 2.19 ± 0.07 | 185.91 ± 4.61 | 5452.62 ± 640.36 | -82.38 ± 2.18 |
| Kv7.2 WT | 2.5 | 18 | -43.78 ± 0.0011 | 3.82 ± 0.24 | 105.41 ± 4.72 | 1889.149 ± 97.66 | -92.30 ± 4.79 |
| p.(N258K) Kv7.2 | 2.5 | 15 | *** -42.91 ±0.0013 | ** 2.93 ± 0.15 | ** 135.65 ± 4.21 | **4550.06 ± 621.66 | -84.78 ± 2.92 |
| p.(G279D) Kv7.2 | 2.5 | 15 | *** -43.17 ± 0.0013 | * 3.19 ± 0.18 | * 127.69 ± 3.38 | **3859.70 ± 254.93 | -85.51 ± 4.13 |
| Kv7.2/Kv7.3WT | 1.25:1.25 | 22 | -44.24 ±0.0019 | 4.26 ± 0.28 | 95.37 ± 4.41 | 1637.05 ± 122.93 | -95.31 ± 4.63 |
| p.(N258K)/Kv7.3 | 1.25:1.25 | 14 | *** -42.76 ± 0.0021 | ***2.78 ± 0.14 | *** 137.32 ± 5.59 | ** 3690 ± 364.38 | -89.83 ± 3.94 |
| p.(G279D)/Kv7.3 | 1.25:1.25 | 15 | *** -43.01 ± 0.0018 | ***3.12 ± 0.14 | * 120.92 ± 5.11 | 3135.04± 181.61 | -90.33 ± 3.61 |
| Kv7.2/p.(N258K)/Kv7.3 | 0.625:0.625:1.25 | 13 | *** -43.06 ± 0.0017 | ***3.08 ± 0.18 | *** 133.06 ± 5.78 | *3337.08 ± 418.03 | -87.27 ± 4.78 |
| Kv7.2/p.(G279D)/Kv7.3 | 0.625:0.625:1.25 | 14 | *** -43.24 ± 0.0021 | *3.34 ± 0.21 | ** 122.64 ± 7.61 | 3092.34 ± 334.87 | -89.78 ± 4.77 |

Data shown represent the Ave ± SEM. Statistically significant differences are indicated by **p* < 0.05, ***p* < 0.01, ****p* < 0.001 based on one-way ANOVA Tukey test.

**
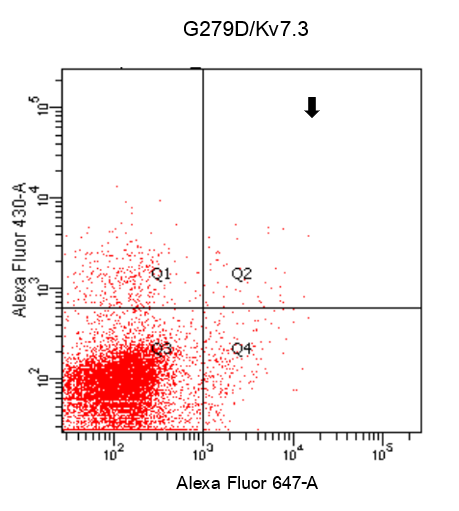

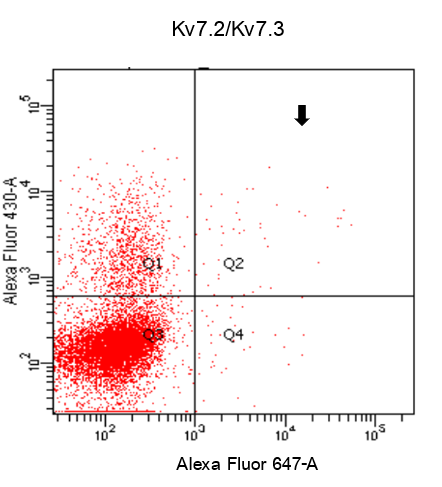

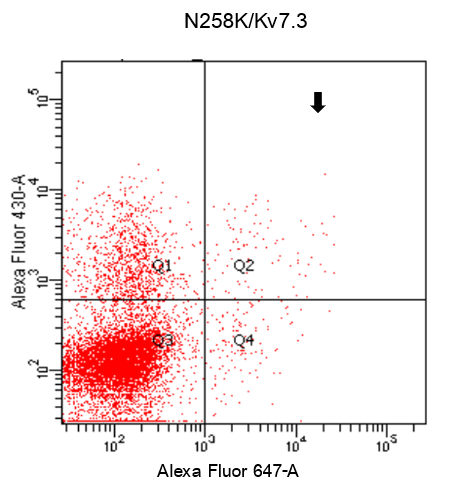

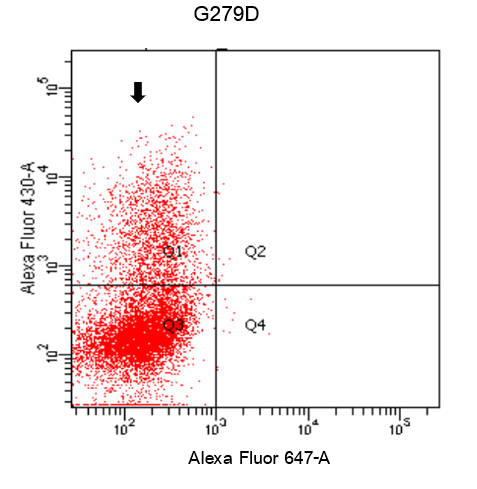

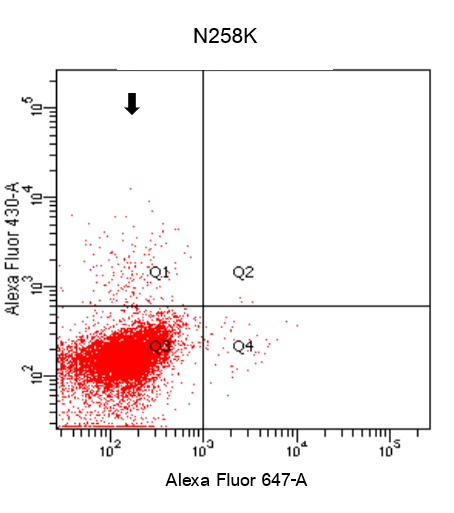

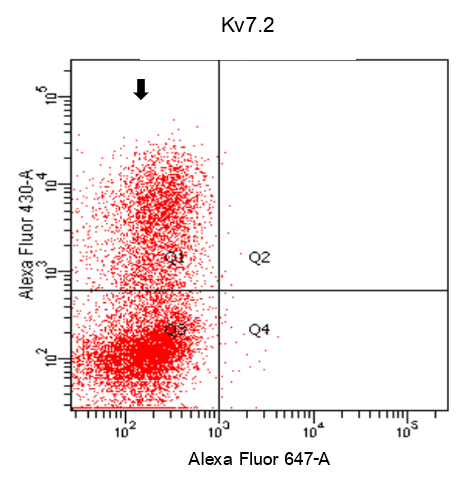

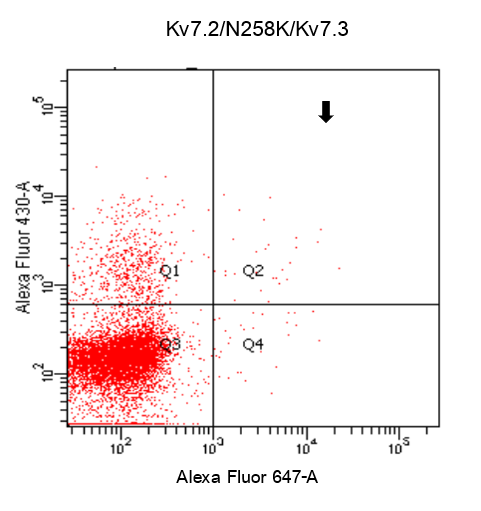

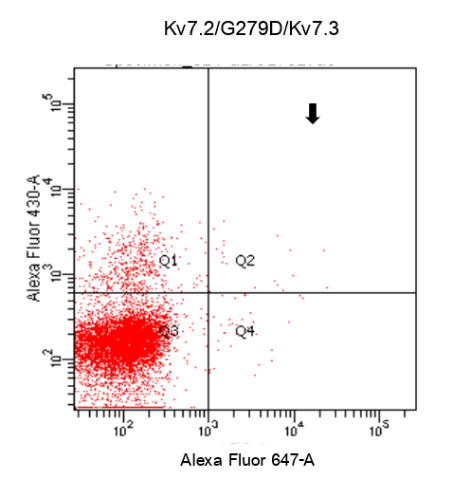
**

**h**

**g**

**f**

**e**

**d**

**c**

**b**

**a**

**Supplementary Figure S1.** **Histogram profiles of sorting gates used for FACS based on Alexa Fluor430 and 647 channels.** Panels (a-c) were used to define KCNQ2-GFP sorting gates, Panels (d-h) were used to define KCNQ2-GFP and KCNQ3-DYKDDDDK tag, conjugated to Alexa Fluor 647 sorting gates. These gates were positioned such that Q1: cells expressed GFP, Q2: cells expressed GFP& DYKDDDDK tag, Q3: non-transfected cell, Q4: cells expressed DYKDDDDK tag.


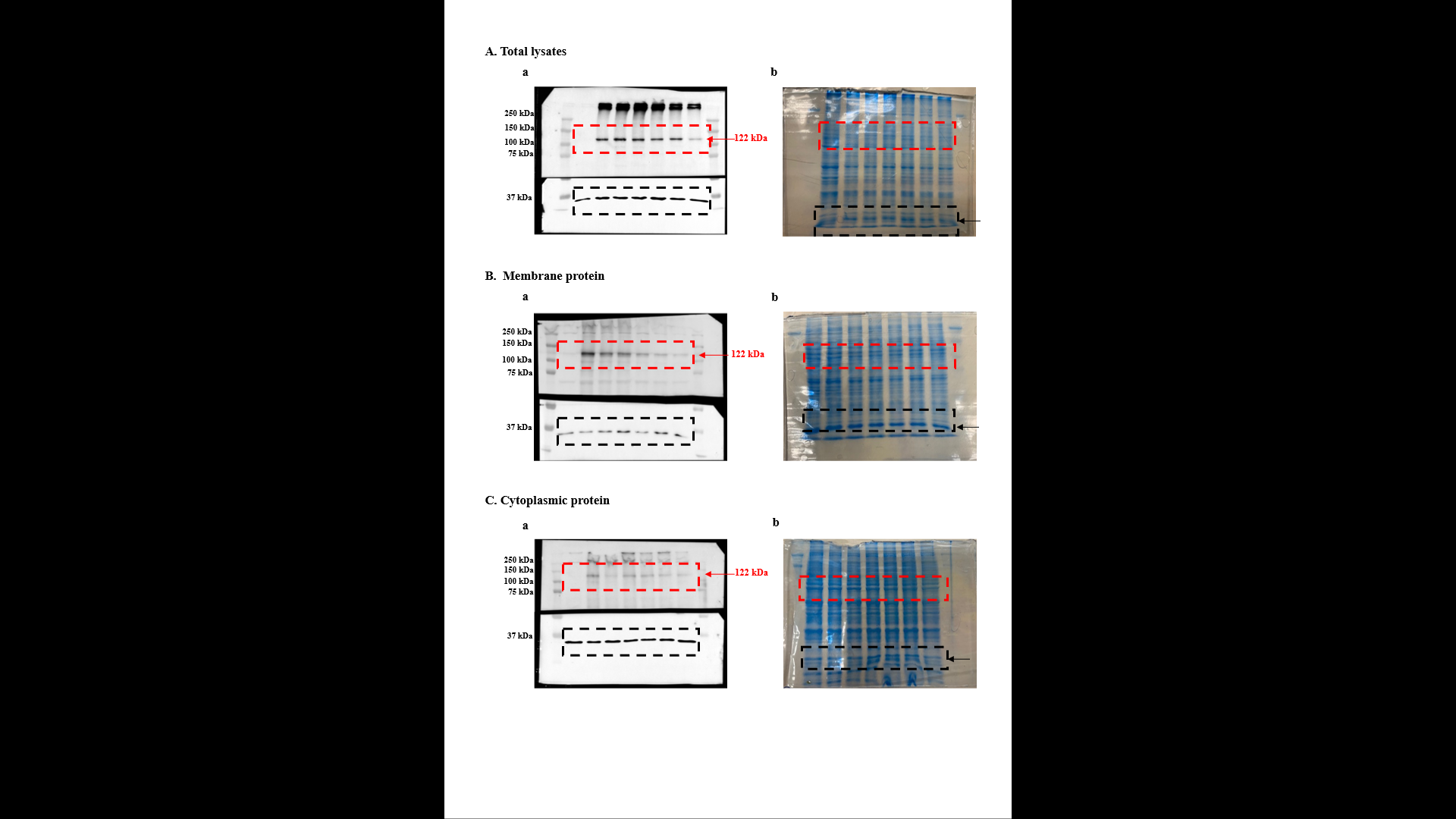


**Supplementary Figure S2.** Original membranes (a) and gels (b) before cropping of dashed-line regions depicted in Figure 5c (A) Total lysate, (B) membrane protein fraction and (C) Cytoplasmic protein fraction. Red arrow indicates Kv7.2-tagged GFP (122 kDa) Black arrow indicates the GAPDH

(37 kDa).


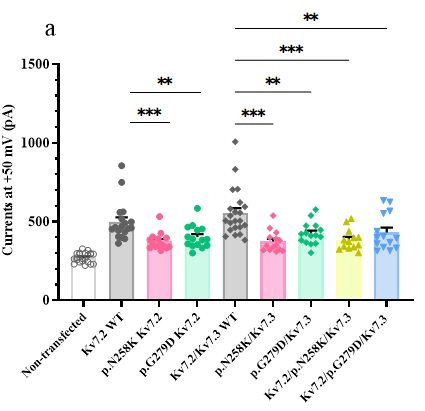


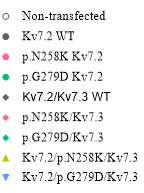

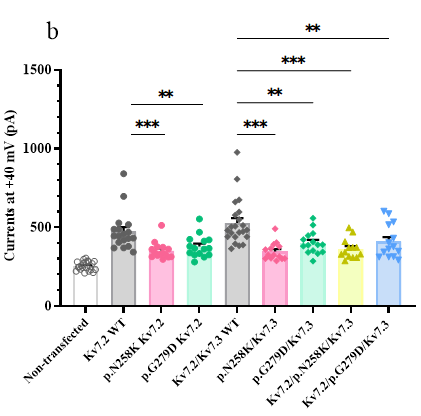


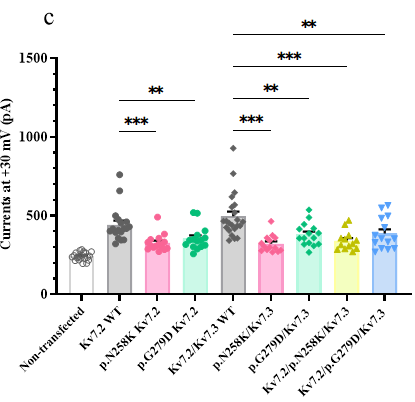

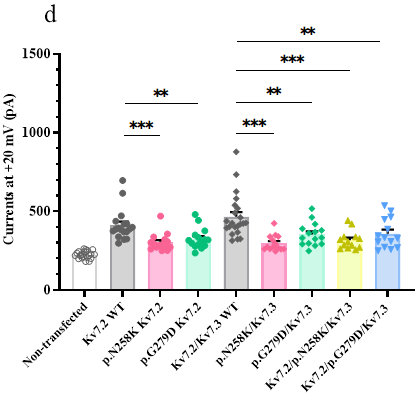


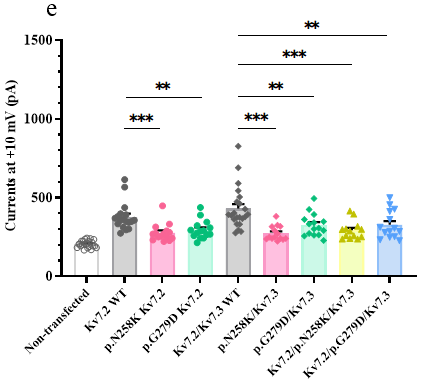

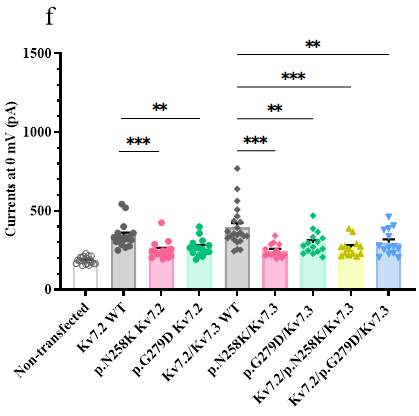


**Supplementary Figure S3. Currents of mutant channels compared with WT at conditional voltage 0 mV to +50 mV.** Statistically significant differences are indicated by **p* < 0.05, ***p* < 0.01, ****p* < 0.001, ns = no significant based on one-way ANOVA Tukey test.


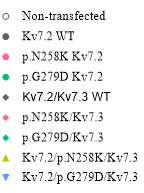

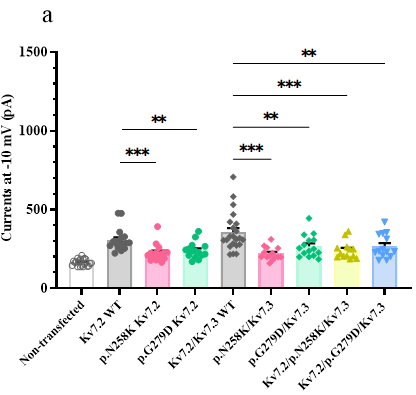

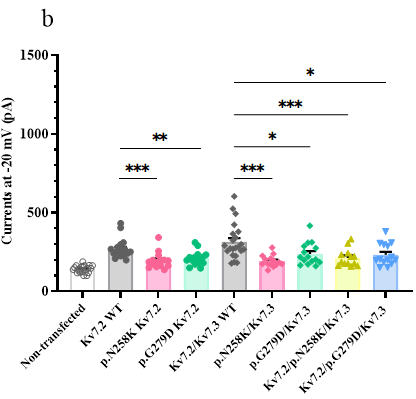


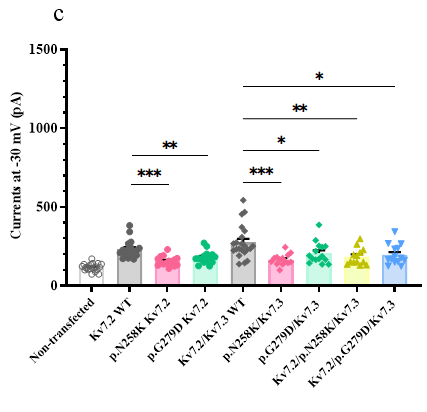

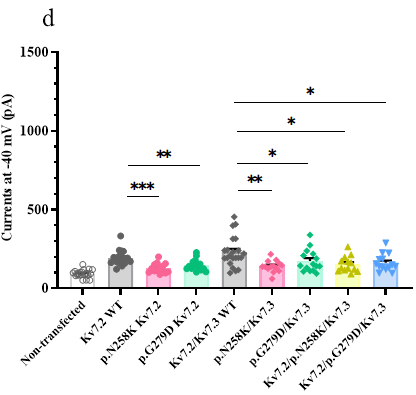


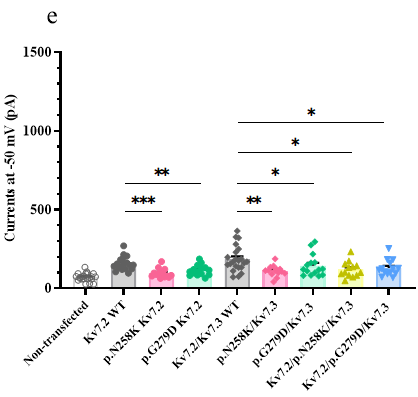


**Supplementary Figure S4. Currents of mutant channels compared with WT at conditional voltage -50 mV to -10 mV.** Statistically significant differences are indicated by **p* < 0.05, ***p* < 0.01, ****p* < 0.001, ns = no significant based on one-way ANOVA Tukey test.

e


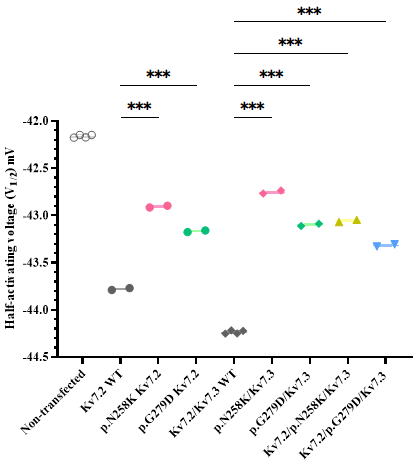

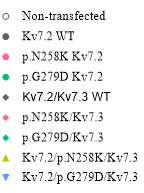


a

c

b


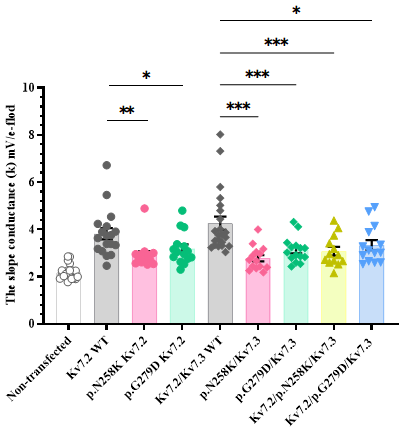


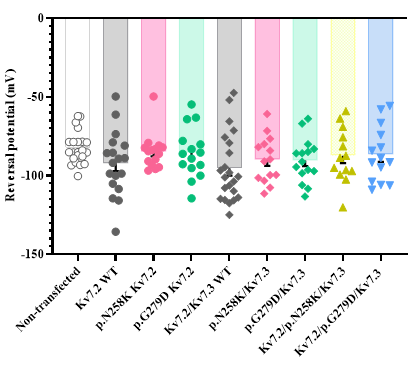


**Supplementary Figure S5.** **Comparison of Ave ± SEM in electrophysiological parameter.**

**(a)**The half-activating voltage (V_1/2_) **(b)** The slope conductance (k**) (c)** Reversal potential detected the equilibrium potential for K+ ion.
